# RNA Polymerase Promotes Splicing, Prevents Degradation

**DOI:** 10.1371/journal.pbio.0040183

**Published:** 2006-05-02

**Authors:** Richard Robinson

As it rolls off the DNA assembly line, RNA faces an uncertain fate. Down one path lies further processing and, ultimately, translation into protein. Down the other, though, lies oblivion—degradation by RNA-digesting enzymes, and recycling back into the nucleotides from which it came. A new study by Martin Hicks, Klemens Hertel, and colleagues shows that efficient processing is promoted, and oblivion avoided, by a close linkage between the machinery of RNA synthesis and that of RNA processing.

Making a protein from a DNA gene requires first making an RNA copy, a process handled by the enzyme complex RNA polymerase II (pol II). This product, called pre-messenger RNA (pre-mRNA) gets a cap and tail, which protect it from exonucleases, enzymes that could otherwise degrade the RNA starting from its ends. They do not protect it from endonucleases, however, which can attack the middle of the RNA strand and render it useless. Endonucleases are prevented from attacking the pre-mRNA when it is bound by the splicing machinery, another enzyme complex that removes noncoding bits, called introns, and splices together the remaining exons.

To investigate whether pol II helps promote binding of the splicing machinery and thus prevent pre-mRNA destruction, the authors compared pol II, found in all animal cells, with a viral RNA polymerase, T7, in otherwise identical cell-free systems. Both generated similar amounts of pre-mRNAs, but spliced mRNAs were created much more efficiently in the pol II system. This might be due either to faster binding of the splicing machinery to the pre-mRNA or to faster processing of the RNA once it is bound. By analyzing the reaction mixture at different time points, the authors found that binding, not splicing, is the key step affected by the presence of pol II.

This suggests that pol II, while it is not part of the splicing machinery per se, somehow increased the ability of the splicing machinery to find and latch on to the pre-mRNA, thereby preventing RNA degradation and ultimately promoting splicing. They found further support for this conclusion by showing that functional splice sites on the pre-mRNA were needed for this effect to occur. Finally, they built a mathematical model of the entire range of RNA interactions, from synthesis to splicing or degradation, and showed that the key variable in determining the relative concentrations of the various RNA species was the affinity of the splice machinery for the pre-mRNA, highlighting the importance of this step in controlling the fate of the RNA.

This study did not address whether pol II and splicing are functionally linked—that is, whether some structural property or dynamic activity of pol II is coupled with the splicing machinery, or, instead, whether the mere proximity of the two ensures efficient transfer of RNA from one to the other. But other studies have highlighted the importance of pol II's C-terminal domain. Located at one end of this molecular behemoth, this region appears to promote other RNA processing events, including addition of the protective cap, and some splicing factors are known to associate with this domain. Further investigation of these interactions may reveal just how intimately linked transcription and splicing are.

## 

**Figure:**
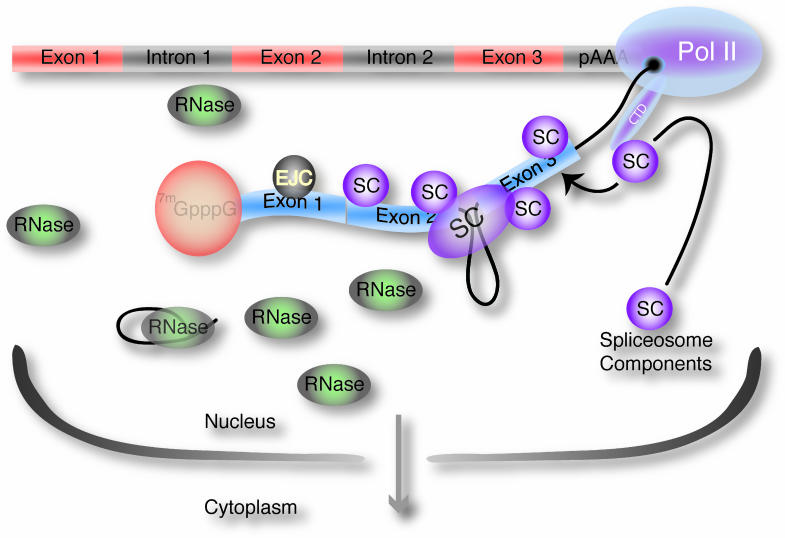
RNA synthesis by Pol II promotes the recruitment of the spliceosome to exon/intron junctions. The rapid association of RNA processing factors with the pre-mRNA prevents its premature degradation.

